# Poorly functioning kidneys with ureteropelvic junction block

**DOI:** 10.4103/0970-1591.33739

**Published:** 2007

**Authors:** Yogesh Kumar Sarin

**Affiliations:** Department of Pediatric Surgery, Maulana Azad Medical College, New Delhi - 110 002, India. E-mail: sarinyk@yahoo.com

Dear Sir,

I read Singh, *et al*.[1] in this journal, with interest.

Rob Pickard and Alan Shindel have already pointed out the weaknesses of the study including its retrospective, nonrandomized nature and short follow-up. To start with, let me seek a clarification- unlike what Pickard commented, I feel that GFRs have been deduced from radionucleotide scans and therefore they relate to the percentage split function. These computer-generated GFR values are not entirely reliable.

The only objective criteria for successful intervention that has been considered is >10% improvement in renal function over baseline. This may not be universally acceptable as we all know that the severely obstructed kidneys to start with may even have >50% split renal function (SRF) that may reduce to <40 % SRF pose a successful surgical intervention. To avoid this apparent paradox, it is pertinent that the authors should have taken up postoperative drainage patterns as one of the criteria. Some of the studies have taken reduction to T 1/2 to <10 min as the strictest criterion. In fact, T 1/2 was not even mentioned as one of the criteria in the diagnosis of ureteropelvic junction block!

Again, defining poorly functioning kidney as one having (computer generated) GFR <25ml/min. may not be universally acceptable. It should rather have been 10ml /min. It would have been interesting to know the surgical outcome in the patient who had preoperative function of 0% SRF.

Again, I wonder about the predictive value of follow-up DTPA scans in context of poorly functioning kidneys. I would also wish to know authors’ opinion in using intravenous pyelogram for postoperative follow-up for ‘poorly-functioning kidneys’.
